# Silencing of miR-17-5p suppresses cell proliferation and promotes cell apoptosis by directly targeting PIK3R1 in laryngeal squamous cell carcinoma

**DOI:** 10.1186/s12935-020-1096-3

**Published:** 2020-01-10

**Authors:** Jian-Xing Wang, Xin-Ju Jia, Yan Liu, Jin-Hui Dong, Xiu-Min Ren, Ou Xu, Sheng-Hui Liu, Chun-Guang Shan

**Affiliations:** 1 Department of Otolaryngology, The Second Hospital of Hebei Medical University, 215 Heping West Road, Shijiazhuang, 050000 People’s Republic of China; 2 Department of Endocrinology, The First Hospital of Hebei Medical University, 89 Donggang Road, Shijiazhuang, 050000 People’s Republic of China; 3 Department of Anesthesiology, The 4th Hospital of Hebei Medical University, 169 Tianshan Street, Shijiazhuang, 050000 People’s Republic of China; 4 Department of Otolaryngology Head and Neck Surgery, The 4th Hospital of Hebei Medical University, 169 Tianshan Street, Shijiazhuang, 050000 People’s Republic of China

**Keywords:** Laryngeal squamous cell carcinoma, miR-17-5p, PIK3R1, Proliferation, Apoptosis

## Abstract

**Background:**

Increasing evidence has suggested that microRNAs (miRNAs) act as key post-transcriptional regulators in tumor progression. Previous studies have confirmed that miR-17-5p functions as an oncogene in multiple cancers and contributes to tumor progression. However, the role and biological functions of miR-17-5p in the development of laryngeal squamous cell carcinoma (LSCC) still remain unknown.

**Methods:**

qRT-PCR was used to detect miRNA and mRNA expression levels in LSCC tissues and cell lines. CCK-8 assay was used to measure cell viability and flow cytometry was performed to evaluate cell apoptosis. Western blot analysis was used to detect the protein levels of BAX, BCL-2, cleaved Caspase-3, PIK3R1 and AKT. Luciferase reporter assay was used to detect the effect of miR-17-5p on PIK3R1 expression. Xenograft animal model was used to test the effect of miR-17-5p on LSCC cell in vivo.

**Results:**

In the present study, we found that miR-17-5p expression level was upregulated in LSCC tissues and cell lines. Depletion of miR-17-5p in LSCC cells significantly reduced cell proliferation and promoted cell apoptosis in vitro and in vivo. Mechanically, knockdown of miR-17-5p in LSCC cells inhibited BCL-2 expression while enhanced BAX and cleaved Caspase-3 protein expression. Moreover, depletion of miR-17-5p in LSCC cells suppressed AKT phosphorylation but did not influence PTEN expression. Importantly, miR-17-5p positively regulated PIK3R1 expression by directly binding to its 3′-untranslated region (UTR). Additionally, PIK3R1, which expression was downregulated in LSCC tissues and cell lines, was involved in LSCC cell survival by modulating the activation of AKT signal pathway. Dysregulation of miR-17-5p/PIK3R1 axis was participated in LSCC cell proliferation and apoptosis by inhibiting the activation of the PI3K/AKT signaling pathway.

**Conclusions:**

In conclusion, our study indicates that the miR-17-5p/PIK3R1 axis plays an essential role in the development of LSCC and provides a potential therapeutic target for LSCC treatment.

## Background

Laryngeal squamous cell carcinoma (LSCC) is the most common head and neck malignancy accounting for more than 95% of head and neck squamous cell carcinoma (HNSCC), with 177,422 new cases and 94,771 deaths worldwide in 2018 [1, 2]. Most of LSCC patients who are diagnosed at early stage may benefit from surgery, followed by radiotherapy and/or chemotherapy [3, 4]. However, the 5-year overall survival rate of patients with LSCC who are asymptomatic in the advanced stage remains lower than approximately 50% [5]. Therefore, an understanding of the driven-element and molecular mechanisms of tumorigenesis in LSCC is crucial.

microRNAs (miRNAs) are the most important post-transcriptional regulators which suppress the expression of protein-coding genes by directly targeting mRNA at the 3′-untranslated region (UTR) for translational repression or degradation [6, 7]. Accumulating studies have shown that miRNAs are implicated in LSCC development, including proliferation, apoptosis, migration and invasion [8–10]. Our previous study has confirmed that miR-486 is involved in LSCC cell migration by targeting FLNA [11]. Moreover, miR-370, which functions as a tumor suppressor, participates in LSCC cell growth by inducing FOXM1 expression [12]. Overexpression of miR-613 reduces LSCC cell proliferation, invasion, and blocks G1/S phase transition by targeting the PDK1 gene [13]. Furthermore, miR-1297, miR-143-3p, miR-503 and miR-205 also promote LSCC cell progression [14–17]. Recent studies have confirmed that miR-17-5p plays critical roles in tumor progression, such as pancreatic cancer, breast cancer, hepatocellular carcinoma, gastric cancer and prostate cancer [18–22]. However, the expression and biological functions of miR-17-5p in LSCC remain unclear.

Increasing evidence has revealed that abnormal activation of PI3K/AKT pathway is associated with the generation of multiple tumors, including LSCC, via regulating cell survival, apoptosis, proliferation, migration, invasion and vesicle trafficking [23–25]. PIK3R1, which encodes the p85α protein, is best known as the regulatory subunit of class 1A PI3Ks through its interaction, stabilization and repression of PI3K-p110 catalytic subunits [26]. PIK3R1 has been identified to be differentially expressed in many human cancers. For example, PIK3R1 functions as a tumor suppressor in hepatocellular carcinomas and renal cancer [27, 28], whereas acts as an oncogene in ovarian and colon tumors and plays a role in tumor progression and metastasis [29, 30]. However, the relationship between PIK3R1 and LSCC cell development has not been fully elucidated.

In the present study, we observed an increased level of miR-17-5p in LSCC tissues and cell lines. Knockdown of miR-17-5p reduced LSCC cell proliferation and induced apoptosis in vitro and in vivo by suppressing the activation of the PI3K/AKT pathway. Importantly, we demonstrated miR-17-5p positively regulated PIK3R1 mRNA and protein expression by targeting its 3′UTR. In addition, PIK3R1 may function as tumor suppressor in LSCC by promoting cell growth. Taken together, our findings indicate that the miR-17-5p/PIK3R1/AKT pathway plays a key role in LSCC proliferation and apoptosis, providing a potential therapeutic target for LSCC treatment.

## Methods

### Patients and samples

39 LSCC samples and non-cancerous adjacent normal tissues were obtained from the Department of Otolaryngology, Second Hospital of Hebei Medical University between September 2017 and July 2018. None of the LSCC patients were treated with radiotherapy or chemotherapy before surgery. All tissues were immediately frozen in liquid nitrogen after surgery and then later stored at − 80 °C for following use. This study was approved by Ethics Committee of Second Hospital of Hebei Medical University. The written informed consent was obtained from every patient. All the experiments in this paper obey World Medical Association Declaration of Helsinki.

### Cell culture and transfection

The human LSCC cell lines (Hep2, SCC-2 and SCC-40) and the normal human oral keratinocyte cell line (HOK) were maintained in our lab. All cells were cultured in RPMI-1640 (Gibco, Beijing, China) with 10% fetal bovine serum (FBS) (Clark Bio, Claymont, DE), 100 units/ml penicillin and 100 μg/ml streptomycin and were incubated at 37 °C in a humidified incubator with 5% CO_2_. The miR-17-5p mimic, mimic-negative control (NC), miR-17-5p inhibitor and inhibitor-negative control (NC) were purchased from GenePharma Co., Ltd (Shanghai, China). Overexpression plasmid of PIK3R1 (pcDNA3.1-PIK3R1) and luciferase reporter vector were purchased from GENEWIZ Company (Suzhou, China). Cell transfections were carried out by using Lipofectamine 2000 (Invitrogen) according to the manufacturer’s protocol.

### Cell proliferation assay

Cell proliferation assay was detected by using a Cell Counting Kit-8 (CCK-8, Dojindo Laboratory, Kyushu, Japan) according to the manufacturer’s protocol. Briefly, LSCC cells were seeded in 96-well plates and then transfected with miR-17-5p inhibitor or pcDNA3.1-PIK3R1 respectively or co-transfected with them both for 72 h. At the indicated times, 10 μl CCK-8 solution was added into each well for another 3 h. Finally, the absorbance was measured at 450 nm by using a microplate reader (Thermo Fisher USA).

### Cell apoptosis analysis

Cell apoptosis was detected by using the Annexin V-FITC/PI apoptosis detection kit (BD Biosciences, USA) following the manufacturer’s instructions. Briefly, after transfection, cells were grown in 6-well plates for 48 h. Then cells were harvested and washed with ice-cold PBS thrice. Then, the cells were stained with Annexin V-fluorescein isothiocyanate and PI. The data analysis was performed using BD FACS Diva software (BD, USA).

### Xenograft animal model

All animal studies were approved by the Institutional Animal Care Committee of Hebei Medical University. 6 weeks’ old BALB/C nude mice were purchased from Vital River Laboratory Animal Technology Co., Ltd. (Beijing, China). 1 × 10^7^ LV-anti-miR-17-5p or LV-anti-miR-NC-infected Hep2 cells were resuspended in 100 μl PBS mixed with 100 μl Matrigel (356234. BD, MA, USA); The suspension of cells was injected subcutaneously into the left dorsal flanks. The volume of xenograft was measured every 3 days. At the end of the experiment, the mice were euthanized by carbon dioxide asphyxiation. The tumor tissues were stored in liquid nitrogen immediately and stored at − 80 °C for western blot or qRT-PCR analysis.

### RNA extraction and quantitative real-time PCR

Total RNA from tissues and cultured cells were extracted by using QIAzol Lysis Reagent (79306) according to the manufacturer’s protocol. For microRNA analysis, the miScripIIRT kit (QIAGEN GmbH, D-40724 Hilden, Germany) was used for reverse transcription, and the miScript SYBR^®^ Green PCR kit was used for qRT-PCR with specific primers for microRNAs. RNU6b (U6) was used as internal control. For mRNA analysis, total cellular RNA was reverse-transcribed to first strand cDNA by using M-MLV First Strand Kit (Life Technologies). And Platinum SYBR Green qPCR Super Mix UDG Kit (Invitrogen) was used for the qRT-PCR of mRNAs. The mRNA expression was normalized to β-actin.

### Luciferase assay

Luciferase assay was performed as previously described [31]. Briefly, Hep2 cells were seeded into a 24-well plate, miR-17-5p mimic (or mimic-NC) was co-transfected with PIK3R1 reporter construct (wild-type or mutant) or the empty vector using Lipofectamine™ 2000 reagent for 48 h. Then the transfected cells were harvested in lysis buffer and detected by Dual-Glo Luciferase Assay System (Promega, Madison, WI) according to the manufacturer’s protocols. Firefly luciferase (FLuc) activity was measured and normalized against the Renilla luciferase (RLuc) activity.

### Western blot analysis

The tissues and cultured cells were lysed with RIPA buffer. The protein was collected for western blot analysis. Equal amounts of protein were run on 10% SDS-PAGE, and electro-transferred to a polyvinylidene fluoride (PVDF) membranes (Millipore). The specific primary antibodies as follows: anti-PIK3R1 (1:1000, abcam, ERP18702), anti-PTEN (1:1000, abcam ab32199), anti-caspase 3 (1:1000, abcam, ab13847), anti-pan-AKT (1:1000, abcam, ab8805), anti-p-AKT (phospho T308) (1:1000, abcam, ab38449), anti-BCL-2 (1:1000, Proteintech, 12,789-1-AP), anti-BAX (1:1000, Proteintech, 50,599-2-Ig) or anti-β-actin (1:1000, Proteintech, 60,008-1-Ig). The memberanes were visualized with Immobilon ECL (Millipore). FusionCapt Advance Fx5 software (Vilber Lourmat) was used to capture the images.

### Target prediction

Potential target genes of miR-17-5p were identified with following miRNA target prediction algorithms: Targetscan (http://www.targetcan.org/mmu_71/).

### Statistical analysis

All of the data were represented as the mean ± S.E.M. Independent Student’s t-test was used for comparisons of differences between two groups. Results were considered statistically significant at *P *< 0.05. Graphpad Prism 7.0 software was using to perform the statistical analysis (GraphPad Software, San Diego, CA, USA).

## Results

### miR-17-5p is upregulated in LSCC tissues and cell lines

**Fig. 1:**
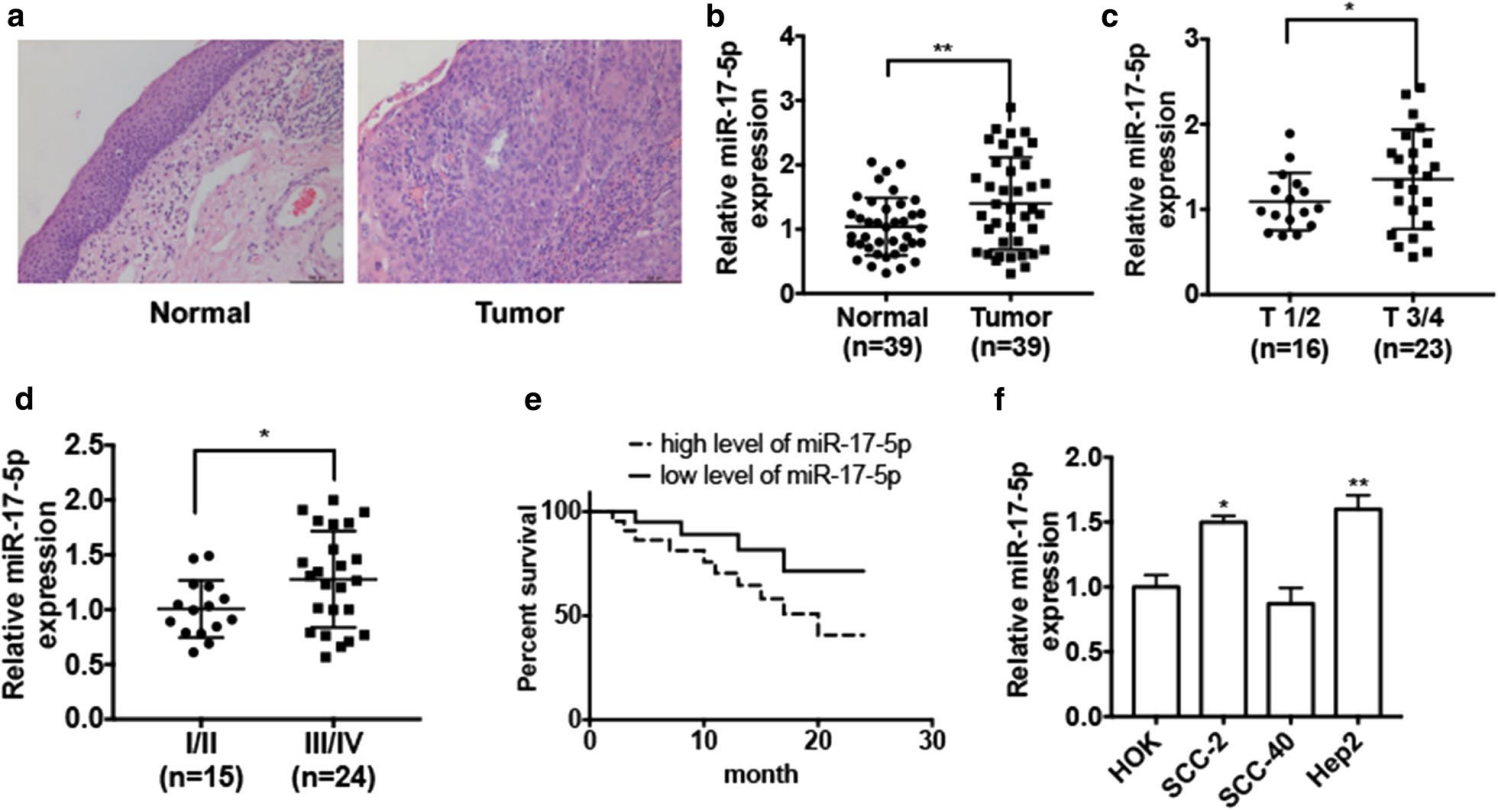
miR-17-5p is upregulated in LSCC tissues and LSCC cell lines. **a** Hematoxylin and eosin staining of normal laryngeal tissues and LSCC tissues. **b** qRT-PCR was used to detected miR-17-5p expression level in LSCC tissues (n = 39) and adjacent normal tissues (n = 39). Normalized to U6. **P < 0.01 vs. normal tissues. **c** qRT-PCR was used to detect the miR-17-5p expression level in the tissues from patients with LSCC in T 3/4 stage (n = 16) and the tissues from patients in T 1/2 stage (n = 23). *P < 0.05 vs. T 1/2 stage. **d** qRT-PCR was used to detect miR-17-5p expression level in tissues from patients with LSCC in TNM I/II stage (n = 15) and in III/IV stage (n = 24). *P < 0.05 vs. TNM I/II stage. **e** Kaplan–Meier analysis was used to analyze the survival rate of LSCC patients with low or high miR-17-5p level. **f** qRT-PCR was used to detect miR-17-5p expression level in three LSCC cell lines (SCC-2, SCC-40 and Hep2) compared with human oral keratinocyte cell line HOK. *P < 0.05, **P < 0.01 vs. HOK cell

To investigate the role of miR-17-5p in LSCC development, we first sought to know the level of miR-17-5p in LSCC tissues. Hematoxylin and eosin staining was used to distinguish the normal laryngeal tissues and the LSCC tissues (Fig. 1a). qRT-PCR result showed that miR-17-5p level was significantly upregulated in LSCC tissues (n = 39) compared with that in adjacent normal tissues (n = 39) (Fig. 1b). We also found that miR-17-5p level was much higher in LSCC tissues from patients with LSCC in T 3/4 stage (n = 16) than that from patients in T 1/2 stage (n = 23) (Fig. 1c). Furthermore, higher miR-17-5p expression levels were maintained in LSCC tissues from patients in III/IV stage (n = 24) than that in II/III stage (n = 15) (Fig. 1d). Importantly, we found that patients with higher expression levels of miR-17-5p in LSCC tissues had poor survival rates compared with the patients who had a lower expression level of miR-17-5p (Fig. 1e). Moreover, we detected the miR-17-5p expression levels in different LSCC cell lines (SCC-2, SCC-40 and Hep2) compared with that in human oral keratinocyte cell line HOK. The result showed that miR-17-5p level was increased in Hep2 and SCC-2 cells but not in SCC-40 cells (Fig. 1f). These results suggest that the miR-17-5p is upregulated in LSCC and may correlated with LSCC progression.

### Depletion of miR-17-5p inhibits cell proliferation and promotes cell apoptosis in LSCC in vitro

**Fig. 2:**
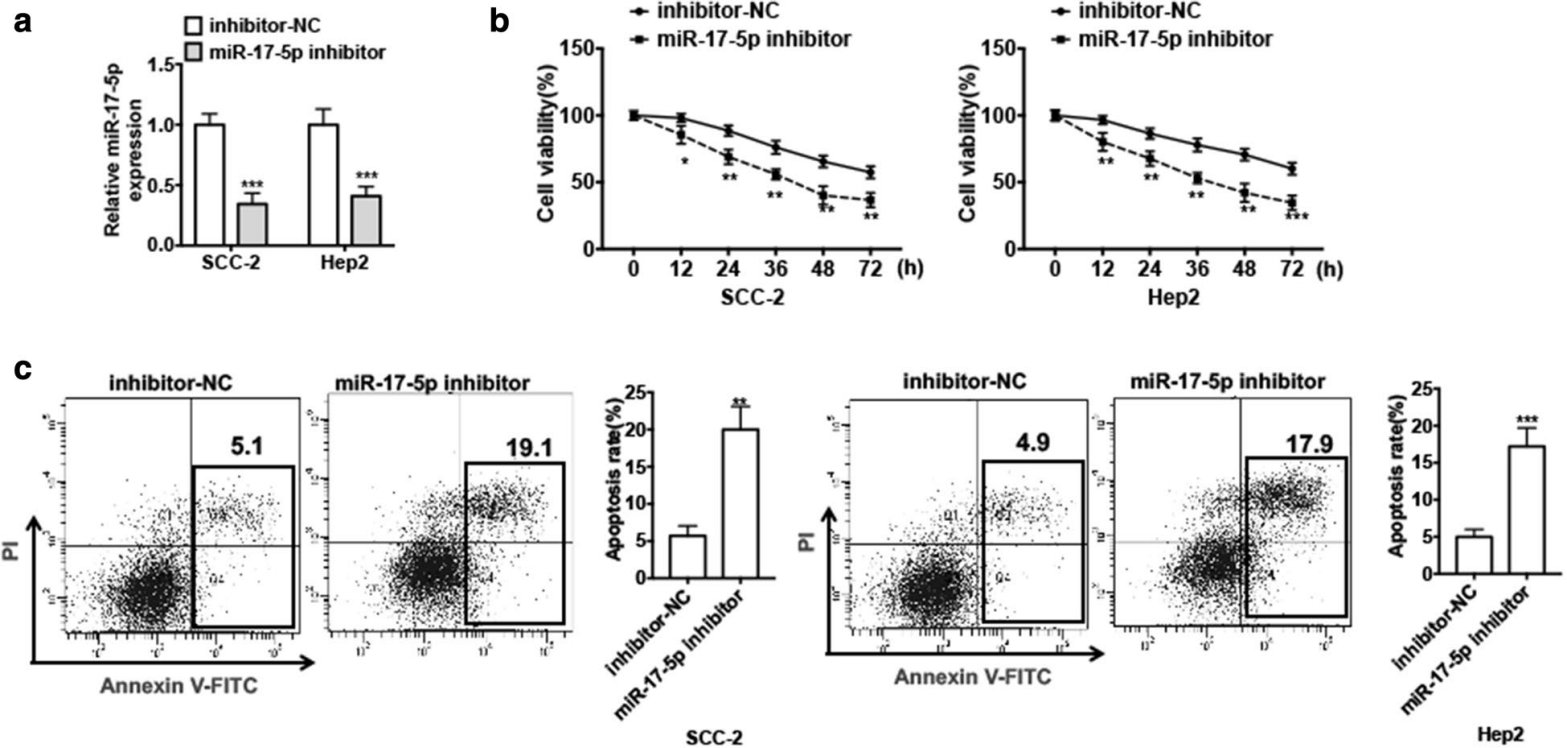
Depletion of miR-17-5p inhibits cell proliferation and promotes cell apoptosis in LSCC in vitro. **a** Hep2 cells and SCC-2 cells were transfected with miR-17-5p inhibitor or inhibitor-NC. miR-17-5p expression was detected by using qRT-PCR. ***P < 0.001 vs. inhibitor-NC. **b** Cells were treated as (**a**), cell viability was measured by using CCK-8 assay in different times. Data shown are mean ± standard deviation of three independent experiments. *P < 0.05, **P < 0.01, ***P < 0.001 vs. inhibitor-NC. **c** Cells were treated as (**a**) for 48 h. Cell apoptosis was assessed by Annexin V-FITC/PI staining. Right panel shows the apoptosis rate of three independent experiments. **P < 0.01, ***P < 0.001 vs. inhibitor-NC

Previous studies have reported that miR-17-5p acts as an onco-miRNA implicated in multiple types of cancers, therefore, we wanted to investigate whether miR-17-5p is involved in LSCC cell survival. First, we transfected miR-17-5p inhibitor or negative control (NC) into Hep2 cells and SCC-2 cells in order to knockdown the miR-17-5p expression in LSCC cells. qRT-PCR results showed that transfection of miR-17-5p inhibitor into LSCC cell lines dramatically decreased the miR-17-5p level compared with negative control (Fig. 2a). Then CCK-8 assay was used to detect cell viability. We found that depletion of miR-17-5p in SCC-2 cells significantly inhibited cell proliferation. This result was further confirmed in Hep2 cells (Fig. 2b). In addition, suppression of miR-17-5p by transfection of miR-17-5p inhibitor in SCC-2 cells and Hep2 cells obviously increased cell apoptosis compared with negative control by flow cytometry-based Annexin V/PI assay (Fig. 2c). These findings reveal that miR-17-5p may act as an onco-miRNA and is involved in cell proliferation and apoptosis in LSCC cells.

### Depletion of miR-17-5p suppresses AKT phosphorylation

**Fig. 3:**
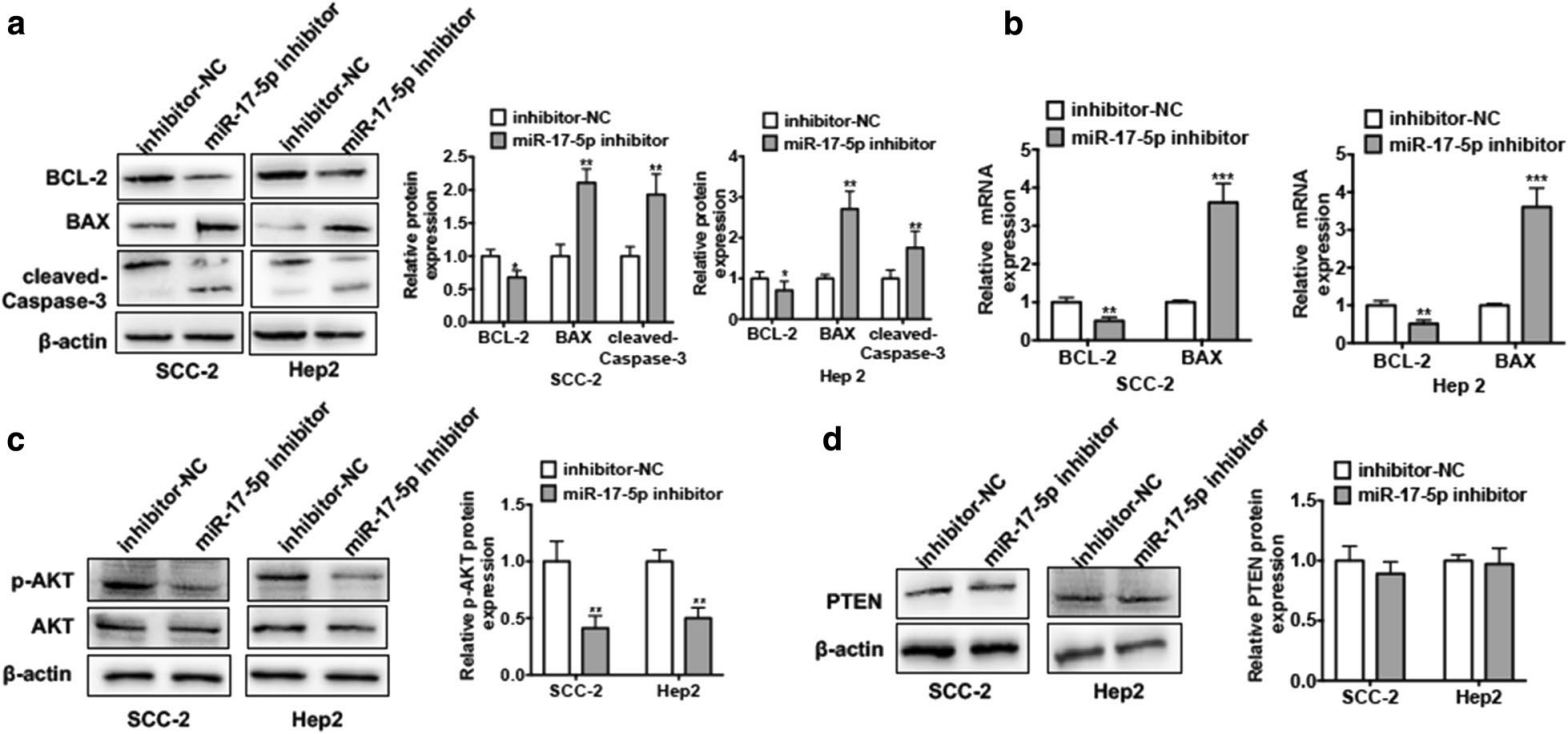
Depletion of miR-17-5p suppresses AKT phosphorylation. **a** Hep2 cells and SCC-2 cells were transfected with miR-17-5p inhibitor or inhibitor-NC. Western blot analysis was used to detect the protein levels of BCL-2, BAX, cleaved Caspase-3. Right panel shows densitometric analysis. *P < 0.05, **P < 0.01 vs. inhibitor-NC. **b** Cells were treated as (**a**), mRNA levels of BCL-2 and BAX were detected by using qRT-PCR. Normalized against β-actin. **P < 0.01, ***P < 0.001 vs. inhibitor-NC. **c** Cells were treated as (**a**), protein levels of p-AKT and AKT were detected by western blot analysis. Right panel shows densitometric analysis. **P < 0.01 vs. inhibitor-NC. **d** Cells were treated as (**a**), PTEN protein level was detected by western blot analysis. Right panel shows densitometric analysis

To further investigate the underlying mechanism of the effect of miR-17-5p on LSCC cell proliferation and apoptosis, we first transfected Hep2 cells and SCC-2 cells with miR-17-5p inhibitor. Western blot analysis was used to measure the protein levels of proliferation maker protein BAX, anti-apoptotic marker BCL-2 and apoptotic maker cleaved Caspase-3. As shown in Fig. 3a, knockdown of miR-17-5p in both cells increased BAX and cleaved Caspase-3 protein levels while reduced BCL-2 protein level. qRT-PCR results also confirmed that transfection of miR-17-5p inhibitor into SCC-2 and Hep2 cells increased BAX mRNA expression level whereas decreased BCL-2 mRNA level (Fig. 3b). Given that BAX and BCL-2 are the important downstream genes of PI3K/AKT pathway, which is involved in cell proliferation, apoptosis and cell cycle, we next investigated whether miR-17-5p regulates BAX/BCL-2 expression by modulating the activation of PI3K/AKT pathway. As shown in Fig. 3c, depletion of miR-17-5p in Hep2 cells and SCC-2 cells suppressed the phosphorylated AKT (p-AKT) protein level. However, the tension homology deleted on chromosome 10 (PTEN) protein level was not changed significantly, indicating that the miR-17-5p may modulate the AKT phosphorylation (Fig. 3d). These results demonstrate that knockdown of miR-17-5p may inhibit AKT phosphorylation and influence the activation of PI3K/AKT pathway.

### PIK3R1 is a direct target of miR-17-5p

**Fig. 4:**
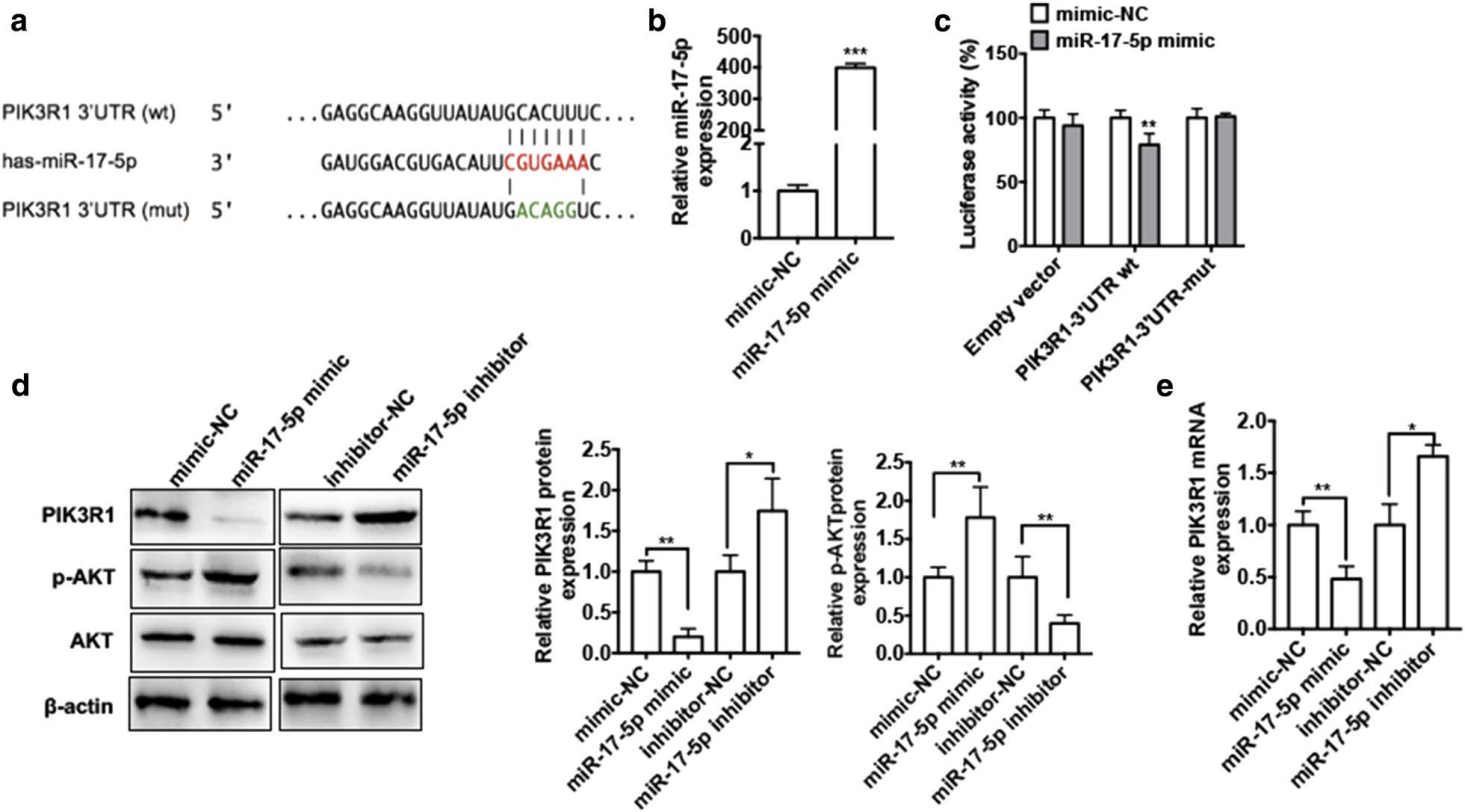
PIK3R1 is a direct target of miR-17-5p. **a** Potential binding site of miR-17-5p at the 3′ UTR of PIK3R1 mRNA. **b** Hep2 cells were transfected with miR-17-5p mimic or mimic-NC. The miR-17-5p expression level was detect by using qRT-PCR. ***P < 0.001 vs. mimic-NC. **c** Hep2 cells were co-transfected with miR-17-5p mimic or mimic-NC and luciferase reporter plasmid containing wild-type or mutated miR-17-5p-binding site (mut) at PIK3R1 3′-UTR. Luciferase reporter assay was used to detect luciferase activity. **P < 0.01 vs. mimic-NC. **d** Hep2 cells were transfected with miR-17-5p mimic, miR-17-5p inhibitor or negative control. Western blot analysis was used to detect protein expression levels of PIK3R1, AKT and p-AKT. Right panel shows densitometric analysis. *P < 0.05, **P < 0.01 vs. corresponding negative control. **e** Cells were treated as (**d**), PIK3R1 mRNA level was detected by using qRT-PCR. *P < 0.05, **P < 0.01 vs. corresponding negative control

To elucidate the underlying mechanism of the regulation of PI3K/AKT pathway by miR-17-5p, we predicted to the target genes of miR-17-5p by TargetScan (http://www.targetscan.org). According to the candidate genes, PIK3R1 aroused our attention. PIK3R1, as the subunit of PI3K, which plays a critical role in the activation of PI3K pathway by binding and stabilizing PI3K p110 catalytic subunit, influenced downstream of AKT and disturbance in the balance between cell apoptosis and proliferation. We found that there exists a putative miR-17-5p-binding site in the PIK3R1 3′-UTR (Fig. 4a). To further confirm the relationship between miR-17-5p and PIK3R1, we constructed dual-luciferase reporters including the wild-type or mutant 3′ UTR of PIK3R1 and assessed luciferase reporter assay. First, we overexpressed miR-17-5p in Hep2 cells with transfection of miR-17-5p mimic. As shown in Fig. 4b, transfection of miR-17-5p into Hep2 cells markedly increased miR-17-5p expression compared with negative control. Then dual-luciferase reporter assay was performed. To do this, Hep2 cells co-transfected with PIK3R1-3′UTR-reporter plasmid containing the miR-17-5p-binding site (wt or mut) and miR-17-5p mimic. As shown in Fig. 4c, miR-17-5p mimic significantly decreased luciferase activity mediated by wild-type 3′-UTR but had no effect on the luciferase activity mediated by its mutant. Next, we performed the loss and gain experiments to determine whether miR-17-5p influence PIK3R1 expression. As shown in Fig. 4d overexpression of miR-17-5p in Hep2 cells obviously reduced while knockdown of miR-17-5p induced PIK3R1 protein level compared with negative control. Constantly, p-AKT protein level was significantly downregulated in the Hep2 cells after transfecting with miR-17-5p inhibitor while upregulated after transfecting with miR-17-5p mimic. In addition, transfection of miR-17-5p mimic in Hep2 cells significantly decreased PIK3R1 mRNA level compared with mimic-NC. Conversely, transfection of miR-17-5p inhibitor could increase PIK3R1 mRNA expression (Fig. 4e). These results show that miR-17-5p negatively regulates PIK3R1 level by directly targeting its 3′UTR.

### PIK3R1 is downregulated in LSCC tissues and cell lines

**Fig. 5:**
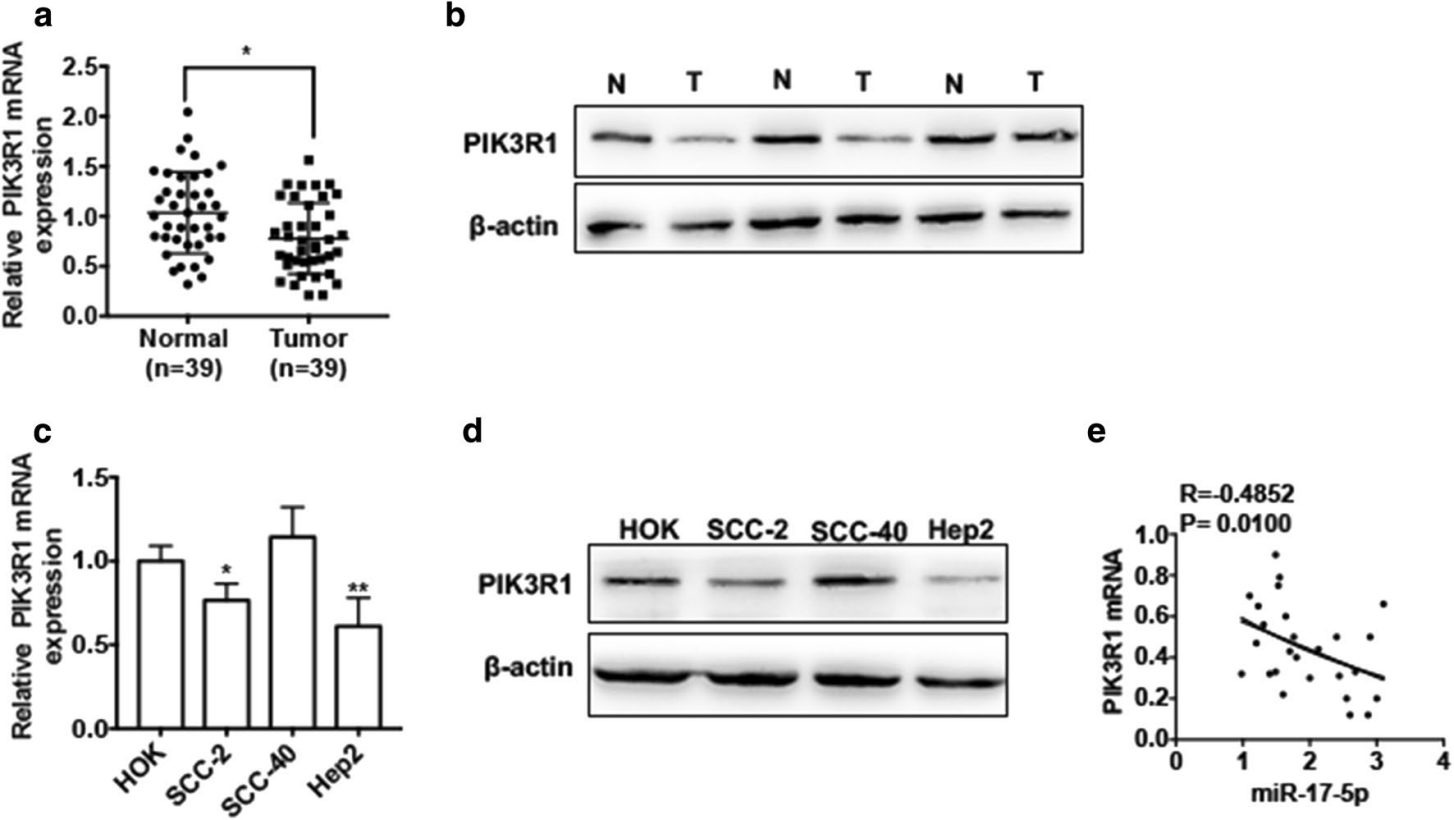
PIK3R1 is downregulated in LSCC tissues and cell lines. **a** qRT-PCR was used to detect the mRNA level of PIK3R1 in LSCC tissues (n = 39) and adjacent normal tissues (n = 39). *P < 0.05 vs. normal tissues. **b** Western blot analysis was used to detect the protein level of PIK3R1 in LSCC tissues and adjacent normal tissues. The representative experiments were present. **c** qRT-PCR was used to detect PIK3R1 mRNA expression in three LSCC cell lines (SCC-2, SCC-40 and Hep2) compared with human oral keratinocyte cell line HOK. *P < 0.05, **P < 0.01 vs. HOK cell. **d** Western blot analysis was used to detect PIK3R1 protein level in three LSCC cell lines (SCC-2, SCC-40 and Hep2) compared with human oral keratinocyte cell line HOK. The representative experiments were present. **e** Pearson correlation was used to analyze the relationship between miR-17-5p and PIK3R1 mRNA (R = − 0.4852, P = 0.0100)

To measure the expression level of PIK3R1 in LSCC, we then examined the mRNA level of PIK3R1 in LSCC tissues. qRT-PCR result showed that mRNA level of PIK3R1 was markedly lower in LSCC tissues (n = 39) than that in normal tissues (n = 39) (Fig. 5a). Similarly, the LSCC tissues had a lower protein level of PIK3R1 than normal tissues (Fig. 5b). Moreover, we measured the expression level of PIK3R1 in different LSCC cell lines compared with that in human oral keratinocyte cell line HOK. As shown in Fig. 5c, the mRNA level of PIK3R1 was decreased in Hep2 and SCC-2 cells but not in SCC-40 cells. Consistently, Hep2 cells and SCC-2 cells had a lower protein level of PIK3R1 (Fig. 5d). Additionally, the correlation analysis also revealed that there was a negative correlation between miR-17-5p and PIK3R1 in LSCC tissues (P = 0.0100, R = − 0.4852) (Fig. 5e). These findings indicate that PIK3R1 expression is reduced in LSCC tissues and cell lines.

### miR-17-5p/PIK3R1 axis plays an essential role in LSCC cell proliferation

**Fig. 6:**
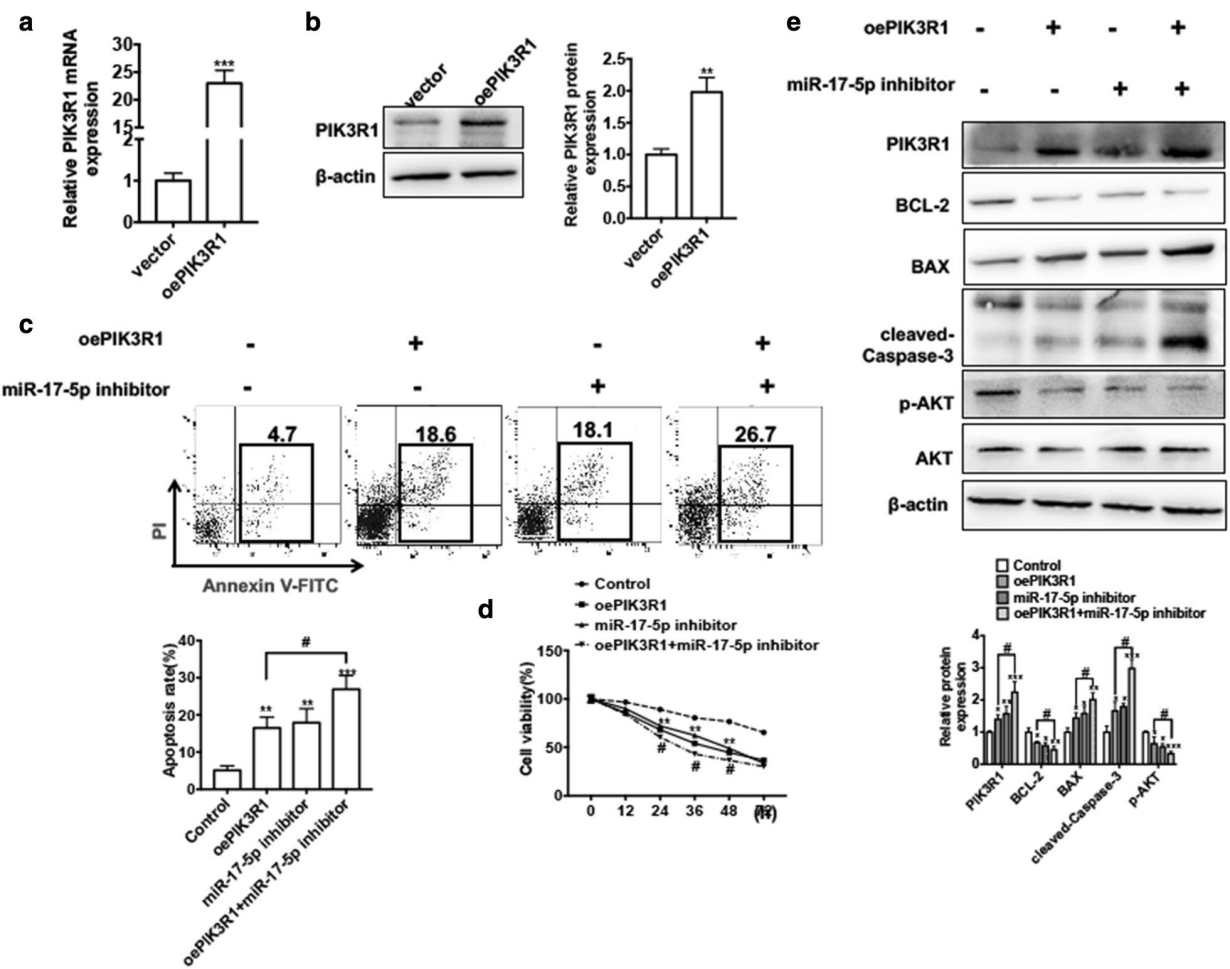
miR-17-5p/PIK3R1 axis plays an essential role in LSCC cell proliferation. **a** Hep2 cells were transfected with pcDNA3.1-PIK3R1 or empty vector. The mRNA level of PIK3R1 was detected by qRT-PCR. ***P < 0.001 vs. empty vector. **b** Hep2 cells were treated as (**a**), the protein level of PIK3R1 was detected by western blot analysis. **P < 0.01 vs. empty vector. **c** Hep2 cells were transfected with pcDNA3.1-PIK3R1 and miR-17-5p inhibitor respectively or transfected them together. Cell apoptosis was detected by Annexin V-FITC/PI staining. Below panel shows the analysis of apoptosis rate. **P < 0.01, ***P < 0.001 vs. corresponding negative control; ^#^*P *< 0.05 vs. pcDNA3.1-PIK3R1. **d** Hep2 cells were treated as (**c**), cell viability was detected by CCK-8 assay in different times. **P < 0.01 vs. corresponding negative control; ^#^*P *< 0.05 vs. pcDNA3.1-PIK3R1. **e** Hep2 cells were treated as (**c**), the protein levels of PIK3R1, BCL-2, BAX, cleaved Caspase-3, p-AKT and AKT were detected by western blot analysis. Below panel shows densitometric analysis. *P < 0.05, **P < 0.01 vs. corresponding negative control; ^#^*P *< 0.05 vs. pcDNA3.1-PIK3R1

To investigate whether miR-17-5p participated in LSCC cell proliferation by regulating PIK3R1 expression, we wanted to know the effect of PIK3R1 in LSCC cell growth. Because Hep2 cells had a much higher PIK3R1 mRNA expression level, we chose it for the following experiment. First, we conducted the overexpression plasmid of PIK3R1 (pcDNA3.1-PIK3R1) and transfected it into Hep2 cells. qRT-PCR and western blot analysis results showed that transfection of pcDNA3.1-PIK3R1 could significantly increase the PIK3R1 protein and mRNA level compared with empty vector, indicating that pcDNA3.1-PIK3R1 had a higher efficiency in increasing expression of PIK3R1 in Hep2 cells (Fig. 6a, b). Then the functional experiments revealed that overexpression of PIK3R1 significantly decreased the cell viability while increased the apoptosis in Hep2 cells (Fig. 6c lane 2 and d). Western blot analysis also showed that overexpression of PIK3R1 in Hep2 cells decreased the protein levels of BCL-2 and p-AKT whereas increased protein levels of cleaved Caspase-3 and BAX (Fig. 6e lane 2). Additionally, we performed the rescued experiments to demonstrate whether PIK3R1 mediating miR-17-5p and LSCC cell proliferation and apoptosis. Apoptosis analysis showed that overexpression of PIK3R1 in Hep2 cells promoted cell apoptosis and this effect could enforced by co-transfected with miR-17-5p inhibitor in Hep2 cells (Fig. 6c). CCK-8 assay result also showed that overexpression of PIK3R1 combination with knockdown of miR-17-5p in Hep2 cell had a much lower growth ability compared with overexpression of PIK3R1 alone (Fig. 6d). Thereafter, overexpression of PIK3R1 in Hep2 cell decreased the protein levels of BCL-2 and p-AKT. This inhibitory effect could be enhanced by co-transfection of pcDNA3.1-PIK3R1 and miR-17-5p inhibitor in Hep2 cells. Consistently, co-transfection of pcDNA3.1-PIK3R1 and miR-17-5p inhibitor in Hep2 cells increased BAX and cleaved Caspase-3 protein level compared with overexpression of PIK3R1 alone (Fig. 6e). Taken together, these findings indicate PIK3R1/miR-17-5p play an important role in LSCC cell apoptosis and proliferation.

### miR-17-5p is involved in LSCC proliferation in vivo

**Fig. 7:**
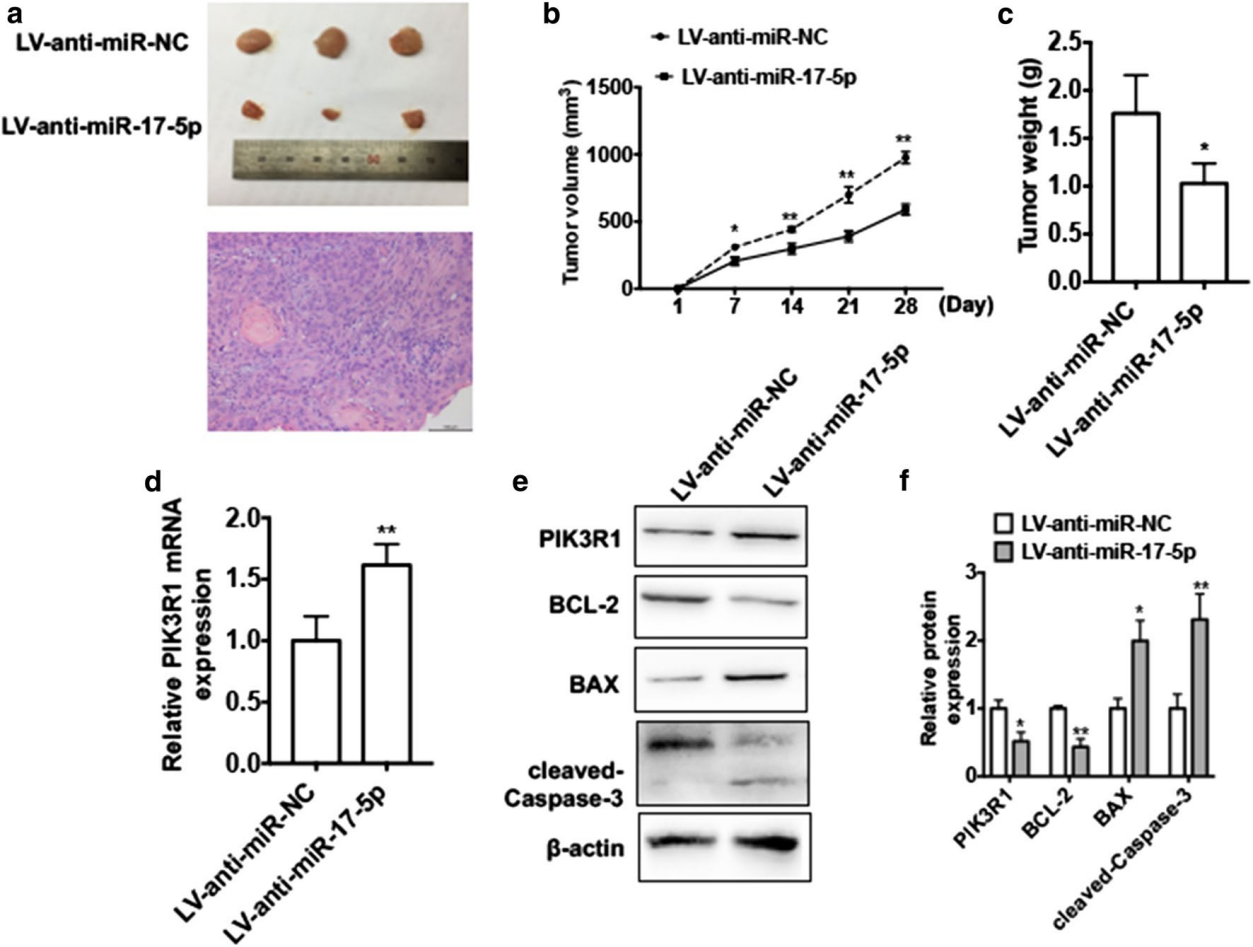
miR-17-5p is involved in LSCC proliferation in vivo. Hep2 cells engineered to stably knockdown of miR-17-5p (LV-anti-miR-17-5p) or negative control (LV-anti-miR-NC). Then cells were injected subcutaneously in 200 μl 1640/Matrigel (100:100) into the left posterior ankle of the nude mice to establish xenograft tumors (each group n = 6). **a** Images of tumors excised from each group of mice at the final time point (28 days after injection) and the hematoxylin and eosin staining of xenograft tumor. **b** Tumor volumes were monitored by direct measurement with calipers and calculated by the formula: (length × width^2^)/2. *P < 0.05, **P < 0.01 vs. LV-ant-miR-NC. **c** Average weight of tumors in nude mice. *P < 0.05 vs. LV-anti-miR-NC. **d** RNA was extracted from excised tumors and the mRNA level of PIK3R1 was detected by using qRT-PCR. **P < 0.01 vs. LV-anti-miR-NC. **e** Total proteins were extracted from excised tumors and the protein levels of PIK3R1, BCL-2, BAX and cleaved Caspase-3 protein were determined by western blotting. Right panel shows densitometric analysis. *P < 0.05, **P < 0.01 vs. LV-anti-miR-NC

To validate the pathophysiological role of the miR-17-5p in LSCC tumorigenesis, we established LSCC nude mouse xenograft model. To do this, we implanted Hep2 cells with stably knocked down of miR-17-5p (LV-anti-miR-17-5p) or normal Hep2 cells into nude mice. The tumor volume was measured every 3 days. As we expected, depletion of miR-17-5p resulted in smaller tumor volumes compared with negative control group (Fig. 7a, b). Consistent with tumor volume, the mean wet weight of the tumors was significantly lower in mice that received miR-17-5p depleted cells than in the control group (Fig. 7c). qRT-PCR result showed that the PIK3R1 mRNA expression level was increased in miR-17-5p-depleted group compared with that in the control group (Fig. 7d). Moreover, western blot analysis demonstrated that knockdown of miR-17-5p significantly downregulated protein level of BCL-2 and was accompanied by an increase in protein levels of PIK3R1, BAX and cleaved Caspase-3 compared with control group (Fig. 7e). These results suggest that knockdown of miR-17-5p inhibits LSCC growth in vivo.

## Discussion

Dysregulation of signaling pathways has been identified to play a crucial role in initiating and maintaining malignant phenotypes of tumors [32]. In the last decade, studies have demonstrated that uncontrolled activation of PI3K/AKT signaling pathway may be the most frequent driving events involved in development and progression of various cancers, including ovarian cancer, cervical cancer, gastric cancer, glioblastoma, hematological malignant and head and neck cancer [32]. PI3K, containing a catalytic subunit (p110) and a regulatory subunit (p85), regulates the downstream phosphorylation of AKT gene, which is involved in promoting cell survival and proliferation by activating the downstream pathways [33]. p85, which is encoded by PIK3R1, plays a critical role in the activation of PI3K/AKT pathway by binding and stabilizing PI3K p110 catalytic subunit [34]. Therefore, aberrations of PIK3R1, such as gene mutations or downregulation, may play a role in different oncogenic mechanisms and influence distinct downstream signaling processes of AKT pathway. For example, PIK3R1 expression level is markedly downregulated in ovarian cancer, lung cancer, prostate cancer, liver, breast and kidney cancers [34]. Moreover, the lower level of PIK3R1 is associated with a poor survival rate in patients with breast cancer [35]. Further, lower mRNA level of PIK3R1 is a high-risk factor in stage I non-small cell lung cancers [36]. Similarly, downregulation of PIK3R1 mRNA expression is involved in migration and invasion of breast cancer cell in vitro [37]. Importantly, silencing of PIK3R1 in breast cancer could enhance the sensitivity to rapamycin [38]. In the present study, we first confirmed that the levels of PKI3R1 mRNA and protein were significantly downregulated in LSCC tissues and cell lines. Functional experiments revealed that overexpression of PIK3R1 in LSCC cells obviously induced cell apoptosis and reduced cell proliferation. Moreover, overexpression of PIK3R1 in LSCC cells influenced AKT phosphorylation and the levels of downstream genes, such as BAX, BCL-2 and cleaved Caspases-3. These findings indicate that PIK3R1 can potentially be a biomarker and a target of treatment in LSCC. However, whether PIK3R1 level correlate with prognosis of patients with LSCC needs to be clarified with more clinical trials.

In addition to the effect of mutations in PIK3R1 that disrupt function, there are other different factors that affect the gene expression of PIK3R1. miRNAs, as a family of non-coding RNAs, have been widely accepted as regulators of PIK3R1 expression at the post-transcriptional level. For example, miR-479-5p directly targets PIK3R1 in gastric cancer cell and increases cell growth in vitro [39]. miR-486-5p, suppresses cell proliferation in non-small cell lung cancer by regulating PIK3R1 expression and modulating AKT pathway activation [40]. Furthermore, PIK3R1 level is reduced by miR-16-5p, which inhibits cell cycle and enhances cell apoptosis by modulating of the PI3K/AKT/NF-KB pathway [41]. Besides, miR-495, miR-106a-5p, miR-455 and miR-15 were directly or indirectly contribute to the regulation of PIK3R1 expression [42, 43]. In the present study, we demonstrated that miR-17-5p could bind to PIK3R1 3′UTR directly. Overexpression of miR-17-5p could effectively decrease while knockdown of miR-17-5p increase the mRNA and protein levels of PIK3R1 and influence AKT phosphorylation. Moreover, protein levels of downstream targets known to play roles in cell proliferation and apoptosis including BCL-2, BAX, and cleaved Caspase-3 which were influenced by overexpression of miR-17-5p. Silencing of miR-17-5p enhanced the effect of PIK3R1 overexpression on cell proliferation and apoptosis. In addition, we found a negative relationship between miR-17-5p expression level and PIK3R1 mRNA level in LSCC tissues, indicating that dysregulation of the miR-17-5p/PIK3R1 regulatory pathway may be associated with LSCC cell survival.

It is well known that one miRNA may regulate different target genes, whereas one gene may be affected by multiple miRNAs. miR-17-5p acts as an oncogene in different cancers by specifically binding to and interfering with different targets. For example, miR-17-5p is overexpressed in gastric cancer tissues and cell lines. Knockdown of miR-17-5p reduces the proliferation and induces the apoptosis in SGC7901 cells by regulating the expression of Early Growth Response 2 (EGR2) [44]. Similarly, miR-17-5p expression is reduced in breast cancer tissues. Overexpression of miR-17-5p inhibits cell proliferation, migration, and invasion by regulating AIB1 expression [45]. Furthermore, miR-17-5p, which is upregulated in pancreatic cancer, functions as an oncogene by directly targeting RBL2 expression [19]. Similar to the previous studies, we demonstrated that miR-17-5p was dramatically unregulated in LSCC tissues and LSCC cell lines. We also revealed that miR-17-5p level was correlated with LSCC T stage and TNM stage, indicating that higher expression may associate with the advanced tumor stage. Importantly, we observed that the higher level of miR-17-5p was associated with poor survival of patients with LSCC. Functionally, we further identified that miR-17-5p acted as an onco-miRNA in LSCC. Knockdown of miR-17-5p in LSCC cells reduced cell proliferation and promoted cell apoptosis in vitro and in vivo. These data suggest that miR-17-5p is a potential therapeutic target and a biomarker of LSCC.

## Conclusion

In conclusion, our findings demonstrate that miR-17-5p is significantly increased in LSCC tissue and cell lines and may potentially be used as a diagnostic biomarker. Higher expression of miR-17-5p correlates with advanced stages of LSCC and a worse prognosis in LSCC. Dysregulation of miR-17-5p is involved in LSCC cell apoptosis and proliferation via directly targeting PIK3R1 3′UTR and modulation of AKT activation. These findings suggest that the miR-17-5p/PIK3R1/AKT axis can be a potential therapeutic target in LSCC treatment.

## Data Availability

Not applicable.

## Abbreviations

LSCC: laryngeal squamous cell carcinoma
PI3K: phosphoinositide 3-kinases
UTR: untranslated region
PI3KR1: phosphoinositide-3-kinase, regulatory subunit 1
PTEN: tension homology deleted on chromosome 10
miRNA: microRNA
RT-qPCR: reverse transcription-quantitative polymerase chain reaction
